# How Culture and Biology Interact to Shape Language and the Language Faculty

**DOI:** 10.1111/tops.12377

**Published:** 2018-09-04

**Authors:** Kenny Smith

**Affiliations:** ^1^ Centre for Language Evolution University of Edinburgh

**Keywords:** Cultural evolution, Gene–culture co‐evolution, Language, Iterated learning

## Abstract

Recent work suggests that linguistic structure develops through cultural evolution, as a consequence of the repeated cycle of learning and use by which languages persist. This work has important implications for our understanding of the evolution of the cognitive basis for language; in particular, human language and the cognitive capacities underpinning it are likely to have been shaped by co‐evolutionary processes, where the cultural evolution of linguistic systems is shaped by and in turn shapes the biological evolution of the capacities underpinning language learning. I review several models of this co‐evolutionary process, which suggest that the precise relationship between evolved biases in individuals and the structure of linguistic systems depends on the extent to which cultural evolution masks or unmasks individual‐level cognitive biases from selection. I finish by discussing how these co‐evolutionary models might be extended to cases where the biases involved in learning are themselves shaped by experience, as is the case for language.

## Abstract 2

Smith gives an excellent overview on research in language evolution, in which he discusses several recent models of how linguistic systems and the cognitive capacities involved in language learning may have co‐evolved. He illustrates how combined pressures on language learning and communication/use produce compositionally structured languages. Once in place, a (culturally transmitted) communication system creates new selection pressures on the capacity for acquiring these systems.

## 1 Introduction

Uniquely among the communication systems of the natural world, human language allows the open‐ended transmission of information: Any idea I am capable of entertaining in my mind can be encoded in a linguistic signal and transmitted to your mind, provided we share a common language. Language achieves this open‐ended expressivity by combining two features seen separately in the communication systems of other animals (Hockett, 1960). First, language exhibits *semanticity*: We use words (or signs in sign languages) and sentences to refer to objects or states of affairs in the world. Second, language is *combinatorial*, at multiple levels—we combine and recombine meaningless contrastive units (speech sounds in spoken language, parameters such as handshape or location in sign languages) to form morphemes, and combine and recombine morphemes to build complex phrases and sentences (e.g., the English sentence *she jumped* includes seven phonemes [transcribed ∫, i, 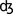, Λ, m, p, t], three morphemes [*she*,* jump*, and the past‐tense morpheme ‐*ed*], and two words). Human languages exploit combinatorial structure to convey complex meanings: All human languages are pervasively *compositional*, in that the meaning of a complex signal is a function of the meaning of its parts and the way in which they are combined (Krifka, 2001). Compositionality allows us to convey differences in meaning by choosing different morphemes to occupy a particular structural position (e.g., *she jumped* means something different from *he jumps* by virtue of the differences in meaning of the elements *she* and *he*, ‐*ed* and ‐*s*), or by combining morphemes in different structural configurations (e.g., *Sam annoyed Jess* means something different from *Jess annoyed Sam*, and the meaning of an ambiguous sentence like *she saw the man with the telescope* depends on the structure one assigns to it). All human languages provide a grammar, a system for combining linguistic units in a rule‐governed way. Knowing the lexicon and grammar of a language allows you to encode your thoughts and (together with the context in which a linguistic expression is produced) decode the encoded thoughts of others.

While semanticity and combinatoriality are found in the communication systems of non‐humans (see e.g., Gill & Bierema, 2013; Townsend & Manser, 2013 for semanticity, Catchpole & Slater, 1995; Payne & McVay, 1971 for combinatorial song, and Ouattara, Lemasson, & Zuberbühler, 2009; Scott‐Phillips, Gurney, Ivens, Diggle, & Popat, 2014; Zuberbuhler, 2002, and Engesser, Ridley, & Townsend, 2016 for cases featuring both semanticity and combinatoriality), no currently attested animal system possesses anything like the open‐ended expressivity of human language.^1^ Explaining the origins and evolution of the uniquely human linguistic system therefore constitutes a major challenge for the cognitive sciences.

The uniqueness of human language suggests some basis in human biology, in the form of uniquely human capacity or predisposition for acquiring and using combinatorial, compositional communication. But the precise form of any individual's linguistic system depends on social learning: we use the language of our linguistic community, and acquire that language through immersion in the rich linguistic environment that community provides. As such, human language is part of the broader phenomenon of human *culture*, where culture refers to any system of social behavior which is transmitted by social learning (Boyd & Richerson, 1985). The form of human language is therefore likely to reflect the interplay of biology and culture, and language evolution likely involves the co‐evolution of the cognitive capacities underlying language and the cultural evolution of languages themselves. Given the pervasive influence of culture on human behavior, many other aspects of human cognition are likely to be products of gene–culture interactions, and language therefore provides a rich domain to explore how cognitive systems are shaped by gene–culture co‐evolution, as well as constituting an important product of those processes. In Section 2, I briefly review the recent experimental literature showing how at least some aspects of linguistic structure can arise from cultural evolution. In Section 3, I look at the implications of this work for theories of the evolution of the human capacity for language.

## 2 Language structure as a product of cultural evolution

**Figure 1:**
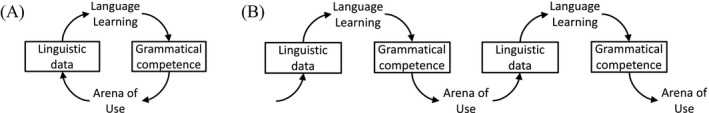
(A) Adapted from Hurford (1990): an individual's grammatical competence is acquired via learning on the basis of linguistic data occurring in the Arena of Use (“The Arena of Use is where communication takes place. It embraces … the kinds of message it is important for us to transmit and receive,” Hurford, 1990, p. 98), and our grammatical competence in turn allows us to produce linguistic data in the Arena of Use. (B) The same cycle unfolded over time: Language use in one individual shapes linguistic competence in other individuals.

Language is transmitted through a repeated cycle of learning and use: We use language in the service of our communicative goals, and we acquire a language by observing language being used in that way by others. We should therefore expect languages to evolve to reflect pressures inherent in language learning and linguistic communication (Hurford, 1990, see Fig. 1). Linguistic variants which are easy to acquire, easy to produce, or useful for communication should proliferate, while those that are hard to learn, effortful to produce, or which don’t serve people's recurring communicative needs will tend to be replaced by better alternatives.

The fact that languages evolve in predictable ways as a result of biases in learning and use forms the basis of the scientific study of language change (e.g., McMahon, 1994; Trask, 1996) and sociolinguistic variation (e.g., Labov, 2010). A growing body of modeling and experimental work seeks to take the same insight and use it to explain not just contemporary variation and change in linguistic systems, but also the origins of fundamental structural properties of natural languages.

This work has its roots in computational models of learning and use in simulated populations (cf. the seminal work by de Boer, 2000; Griffiths & Kalish, 2007; Hurford, 1989; Kirby, 2002; Oudeyer, 2005; Steels, 1998; Wedel, 2012). More recently, experimental *iterated learning* techniques have been developed to study the same processes in the laboratory. This experimental literature has developed from two rather distinct sources, although recent years have seen a welcome confluence of these approaches. One approach builds on work on the development of local conventions during natural language interaction (e.g., Clark & Wilkes‐Gibbs, 1986; Garrod & Anderson, 1987; Krauss & Weinheimer, 1964) and explores how those same interactive processes shape the evolution of novel communication systems (e.g., Fay & Ellison, 2013; Galantucci, 2005; Garrod, Fay, Lee, Oberlander, & MacLeod, 2007). A complementary approach explores what happens when participants are trained on a novel linguistic system (e.g., a miniature language) and then reproduce that language, their reproduction forming the input for learning by subsequent individuals (e.g., Kirby, Cornish, & Smith, 2008; Verhoef, Kirby, & de Boer, 2014); this approach grew out of independent experimental literatures examining the effects of transmission on non‐linguistic behaviors (e.g., Bartlett, 1932; Caldwell & Millen, 2008) and biases in the learning of miniature linguistic systems (e.g., Culbertson, Smolensky, & Legendre, 2012; Hudson, Kam, & Newport, 2005). Recent work has seen these distinct approaches, focusing on interaction and transmission, respectively, combined such that, for example, the products of communicative interaction become the target for learning in others (e.g., Caldwell & Smith, 2012; Kirby, Tamariz, Cornish, & Smith, 2015; Theisen‐White, Kirby, & Oberlander, 2011).

### 2.1 Learning, use, and the emergence of compositionality

These iterated learning and interactive techniques have been applied to study the emergence of symbols (Caldwell & Smith, 2012; Fay & Ellison, 2013; Garrod et al., 2007; Sulik, 2018), combinatoriality (Little, Eryilmaz, & de Boer, 2017; Roberts, Lewandowski, & Galantucci, 2015; Verhoef et al., 2014), and compositionality (Beckner, Pierrehumbert, & Hay, 2017; Kirby et al., 2008, 2015). Following the emphasis in the Introduction on compositionality as a unique feature of human language, here I focus on work on the cultural evolution of compositional structure.

**Figure 2:**
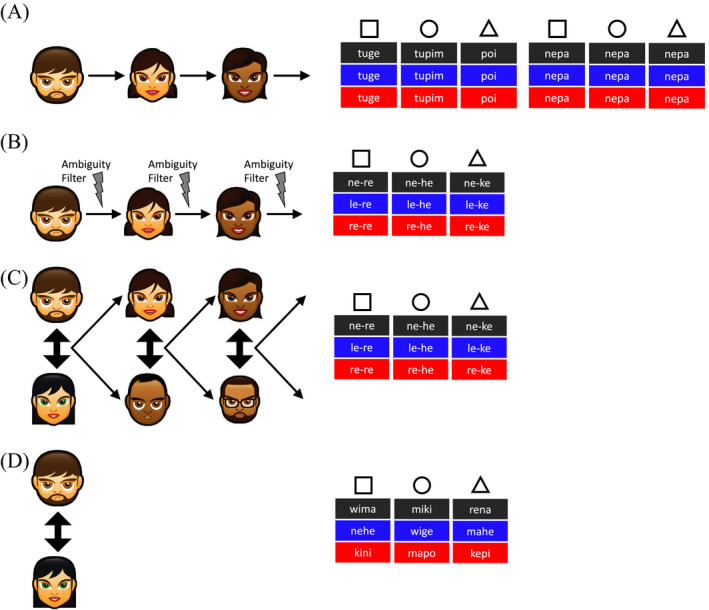
Various experimental paradigms used to model the cultural evolution of language (left) and schematic illustrations of the types of linguistic systems they yield (right). In the illustration of experimental designs, thin arrows indicate intergenerational transmission of the language, and thicker double‐headed arrows indicate communicative interaction. In the schematic languages, we assume labels are used to describe colored shapes: columns give all labels for a given shape, cell colors indicate the color of that shape, and the text in the cell gives the label for the referent with that combination of shape and color; for example, in the first language shown in A, *tuge* is the label used for black, blue, and red squares. Transmission chains based on earning and recall (A) lead to underspecified or degenerate languages (Kirby et al., 2008; Silvey et al., 2015). Transmission chains with filtering of ambiguity (B) or where the data produced during communicative interaction by one pair forms the input to learning in the next pair (C) lead to compositionally structured languages (Beckner et al., 2017; Kirby et al., 2008, 2015). Finally, interaction without transmission (D) results in holistic mappings being maintained (Kirby et al., 2015, but see also discussion in Section 2.2).

In a simple paradigm where participants were asked to learn and then recall a miniature linguistic system (see Fig. 2A), an initial language which provided a distinct idiosyncratic label for each object (a *holistic* language) changed as it was passed from person to person, shedding labels and rapidly losing the ability to encode distinctions (Experiment 1 in Kirby et al., 2008; Silvey, Kirby, & Smith, 2015). In the process of degenerating, these linguistic systems transition through increasingly stable intermediate points, for example, organizing a small number of labels in such a way that they are underspecified for meaning and pick out coherent groups of concepts; however, the eventual end‐point of this repeated process of transmission is often a system where all distinctions are lost.

Learning contains a bias toward simplicity (Brighton, 2003; Culbertson & Kirby, 2016; Kirby et al., 2015): linguistic systems which are simpler (i.e., which have labels which pick out related groups of referents or just fewer distinct labels) are more compressible (i.e., permit shorter, more concise mental representations) and are therefore easier to learn. The assumption that learning involves a search for simple representations has been suggested as a universal bias in cognition (Chater & Vitanyi, 2003), is consistent with cross‐linguistic observations on the acquisition of natural languages (see e.g., Slobin, 1977), and has been proposed as an important factor in shaping linguistic systems (e.g., the kinship, color, and numeral systems of natural languages are among the simplest possible configurations for a given level of communicative utility: see Kemp, Xu, & Regier, 2018, for review). In the experiments illustrated in Fig. 2A, this pressure for simplicity inherent in learning is the sole factor acting on languages as they are passed from individual to individual, and consequently, those languages become extremely simple. In natural languages, this drive toward simplicity is counteracted by language use for *communication*. Using a language for communication, rather than simply attempting to recall it for the purposes of completing an experiment, introduces a pressure for *expressivity*, the potential to encode distinctions which would allow an interlocutor to recover an intended meaning from a signal.

In subsequent work, we introduced a pressure for expressivity, either by imposing an artificial proxy for the requirement in communication to convey distinctions (by artificially filtering out ambiguous labels as the language passed between generations, see Fig. 2B and Experiment 2 of Kirby et al., 2008) or using actual communication between participants to induce a more naturalistic pressure for expressivity, with the language produced during interaction by one pair being used as the input to learning for another pair (Kirby et al., 2015; Winters, Kirby, & Smith, 2015, see Fig. 2C). These experiments and a subsequent more adequately powered replication (Beckner et al., 2017) show that, when the language is under pressure to be not only compressible (as a result of transmission to new learners) but also expressive (either because it is actually being used for communication, or because ambiguity is filtered out), compositional structure gradually evolves (Beckner et al., 2017; Kirby et al., 2008, 2015). In contrast, when the language is used repeatedly for communication by a single pair of participants but not transmitted to naive learners (see Fig. 2D), compositional structure does not evolve; the language remains largely holistic, communicatively functional for individuals well acquainted with that system but (irrelevantly) challenging for naive individuals to learn (Kirby et al., 2015). This work therefore suggests that compositional structure in natural language provides a means of satisfying partially competing pressures from language learning (be simple) and language use (be expressive)—compositionality provides the simplest possible system with the desired degree of expressivity.

### 2.2 Use in learning, learning in use?

Ongoing work suggests a more complex picture for the relative roles of learning and use in shaping linguistic systems. First, a bias toward simplicity may not be the only pressure operating in learning: There may be cases where learners restructure linguistic systems such that they would be better suited for efficient communication (e.g., by case‐marking only where necessary to avoid ambiguity: Fedzechkina, Jaeger, & Newport, 2012; Fedzechkina, Newport, & Jaeger, 2016). If language learning does contain biases toward communicative efficiency, this naturally raises the question of where such biases originate, which the co‐evolutionary modeling discussed in the next section may speak to.

Second, there is some evidence that interaction can also introduce pressures favoring compressibility. Communicative interaction involves rapidly updating one’s beliefs about the (local) linguistic system based on the current interaction; these local adaptation effects result in phenomena such as the rapid emergence of lexical pacts (Clark & Wilkes‐Gibbs, 1986). In Kirby et al. (2015) we showed, using a computational model, that the effect of learning biases in favor of simplicity are apparent even in communication‐only models (illustrated in Fig. 2D) when the pressure for successful, unambiguous communication is reduced, or the role of learning during interaction is increased; in particular, compositionally structured linguistic systems can emerge in communication‐only conditions when learners have seen little linguistic data prior to interaction, or when their behavior is influenced only by their last few interactions. Consistent with this modeling prediction, Winters, Kirby, and Smith (2018) show that participants given minimal training on a miniature linguistic system prior to interaction will, during interaction, restructure that system in ways that are consistent with simplicity preferences, with the details of the systems that emerge depending on the expressivity requirements of the communicative task. Interaction also brings its own constraints, which can resemble simplicity‐based preferences in learning. The tendency to be primed by one's interlocutor during interaction can lead interacting pairs to converge on systems which are less variable (i.e., simpler) than those which would be produced in asocial recall (Fehér, Wonnacott, & Smith, 2016). Indeed, the mere fact of interaction seems to lead people to behave more predictably and regularly, a tendency which is seen in both linguistic (Fehér, Wonnacott, & Smith, 2016) and non‐linguistic (Vesper, van der Wel, Knoblich, & Sebanz, 2011) coordination tasks. Understanding exactly what biases operate during learning and use, and their relative weighting, will provide increasingly fine‐grained insights into the cultural evolution of linguistic systems.

## 3 Implications for the evolution of the capacity for language and constraints on language learning

As reviewed in the previous section, there is a substantial body of work showing how linguistic systems are reshaped during their learning and use, and evidence that this process of cultural evolution might be responsible not only for contemporary language change but also, at much greater time‐depths, for fundamental structural properties of language. This work has important implications for understanding the evolution of the human capacity for language, including what constitutes the capacity for language and how evolution may have shaped it.

### 3.1 The evolution of the capacity for culture

One approach to understanding the relationship between culture and biology in shaping language is to ask what cognitive capacities the cultural evolution of linguistic structure depends on. We could view these as prerequisites that biological evolution must deliver, which set in motion a subsequent process of cultural evolution.

First and most obviously, cultural evolution depends on the capacity for social learning, which is widespread in the animal kingdom. More specifically, the cultural evolution of a compositional linguistic system depends on the ability to learn the form of signals from input, to recognize that those signals are motivated by complex internal or environmental stimuli (i.e., there is a communicative intention that the signal conveys), and to learn a compositional mapping relating signals and communicative intentions. As reviewed in Smith (2018), perhaps surprisingly these capacities are not unique to humans. The capacity to learn and produce complex signals in vocal or other modalities is found in a number of species (Catchpole & Slater, 1995; Garland, Rendell, Lamoni, Poole, & Noad, 2017; Savage‐Rumbaugh, McDonald, Sevcik, Hopkins, & Rubert, 1986). Evidence for the capacity of non‐human animals to reason about mental states, that is, to infer communicative intentions, is patchier (see Scott‐Phillips, 2014, for review), although recent evidence suggests that we may have underestimated the capacity of our closest relatives to reason about others’ minds (Krupenye, Kano, Hirata, Call, & Tomasello, 2016; Schel, Townsend, Machanda, Zuberbühler, & Slocombe, 2013). Mechanisms of associative or reinforcement learning may be all that is required to learn (non‐compositional) inventories of word–object pairings, as evidenced by word learning in dogs (Kaminski, Call, & Fischer, 2004; Pilley & Reid, 2011). Dogs can also be trained to respond appropriately to two‐ and three‐word instructions, where doing so requires comprehension of the semantics of word combination, for example, understanding the difference between *take the bone to the ball* and *take the ball to the bone* (Pilley, 2013; Ramos & Ades, 2012). Captive dolphins and human‐reared chimpanzees and parrots have been trained to respond correctly to more complex compositional commands (Herman, Richards, & Wolz, 1984; Pepperberg, 1981; Savage‐Rumbaugh et al., 1986), although to date few studies have explored the acquisition of compositional meaning‐signal mappings in tightly controlled conditions (but see Medam & Fagot, 2016, for a notable exception).

While these various capacities in non‐humans seem limited in comparison to the corresponding human traits, they suggest that the last common ancestor of humans and our closest non‐linguistic relatives may have had some competence in all of the requisite areas; in other words, evolution may only have elaborated existing capacities in the ancestral population, rather than creating them de novo. We can also scaffold experimental cultural systems in other animals and see how those cultural systems evolve. We found that systematically structured behaviors evolved through transmission in a captive population of baboons (Claidière, Smith, Kirby, & Fagot, 2014); when we provided a behavior that could develop structure (sets of visual patterns), and facilitated transmission of these behaviors from animal to animal, the perceptual and cognitive biases of baboons were sufficient to drive the evolution of structured behaviors. This suggests that, if evolution produced the capacity for cultural transmission of communicative behaviors in our ancestors, interesting types of structure might have developed fairly rapidly even with minimal changes to the rest of the cognitive architecture.

What selection pressures drove these various capacities to a level sufficient to sustain the emergence of a culturally transmitted proto‐linguistic system in our ancestors? One possibility is that these capacities were elaborated through direct selection for the capacity to communicate, for example, due to payoffs associated with the ability to exchange social information (Dunbar, 1996) or acquire complex tool‐making skills (which might be facilitated by language: Morgan et al., 2015); alternatively, they may reflect domain‐general skills that evolve under selection for the more general capacity to learn socially or navigate a complex social world. Another possibility is that they emerge as a by‐product of relaxation of selection in “self‐domesticated” social species (Deacon, 2010; Kirby, 2017; Okanoya, 2015; Thomas & Kirby, 2018). Under any of these scenarios, the emergence of a culturally transmitted communication system might in turn generate new selection pressures for increasingly sophisticated capacities to acquire such systems. It is this co‐evolutionary process we turn to next.

### 3.2 Gene–culture co‐evolution for language

#### 3.2.1 Cultural niche construction in language evolution

Culturally transmitted behaviors can alter the selection pressures acting on an organism's genes, a process known as *cultural niche construction* (Odling‐Smee, Laland, & Feldman, 2003). Culturally transmitted behaviors can shield the genes from environmental pressures which would otherwise result in natural selection and biological adaptation. For instance, technology has allowed humans to inhabit a wide range of hostile environments (from the tropics to the arctic circle) with only relatively modest biological adaptations; culturally transmitted technologies insulate us from our environment, providing adaptations which would otherwise have to be made by biological evolution selecting on available genetic variation.

Cultural practices also create *new* selection pressures which our genes must adapt to. Celebrated examples include dairying, where the culturally transmitted practice of consuming raw animal milk sets up selection for the genes encoding the ability to digest lactose later in life (Gerbault et al., 2011), and yam cultivation, which in the “right” environment generates selection for resistance to malarial parasites that are transmitted by mosquitoes which thrive in environments deforested for agriculture (Durham, 1991). While niche construction is not unique to humans, it may be a particularly powerful force in our evolution, given how pervasively our species construct the environments to which our genes adapt.

**Figure 3:**
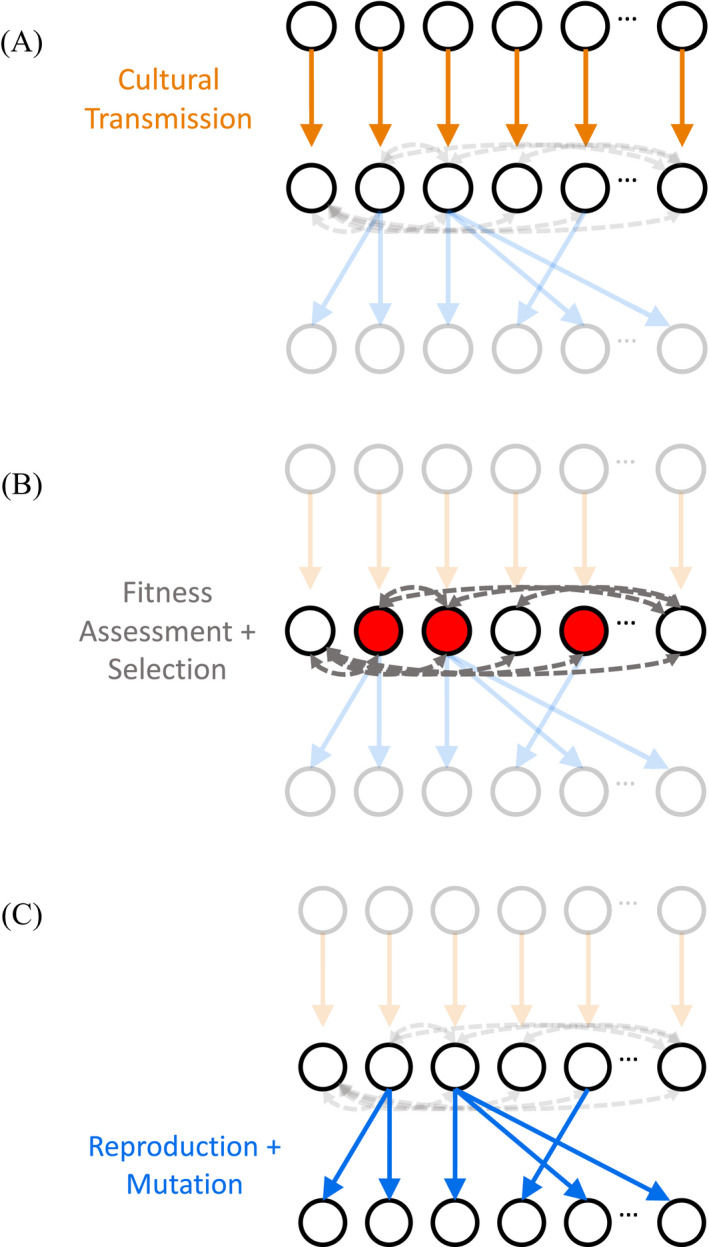
The general schema of a co‐evolutionary model. A population consists of a series of generations (here, 3), each composed of multiple individuals (represented as circles). The population at generation *n* − 1 produces linguistic data to be learned from by the population at generation *n* (A). The fitness of the new population is assessed (e.g., by evaluating their success at communicating with each other, or their speed of learning), and the most successful individuals are selected to reproduce (B). Those individuals then pass on their genes to create a new generation of the population (C), and the process repeats, with generation *n* producing linguistic data for generation *n* + 1. In de Boer (2016), individuals additionally adapt their language system through interaction with their peers. In Chater et al. (2009) and de Boer and Thompson (2018), the target of learning is specified by the experimenter, rather than coming from data produced by the previous generation.

A culturally evolving linguistic system might trigger similar co‐evolutionary processes, and in particular, generate new selection pressures operating on the genes encoding language‐relevant capacities (Deacon, 1997). De Boer (2016) explores this process using a simulation model of co‐evolution (see Fig. 3); while this model is framed as a model of the evolution of vowel systems, his results should apply more broadly. In de Boer's model, individuals learn the vowel system of their parents, then adapt that vowel system during their lifetime, adjusting it to maximize communicative success with other members of their population (which requires vowel discriminability), before passing on their vowel system to their offspring. Under this scenario, cultural evolution drives vowels in a system of fixed size to be maximally dispersed to maximize communicative utility (de Boer, 2000).

**Figure 4:**
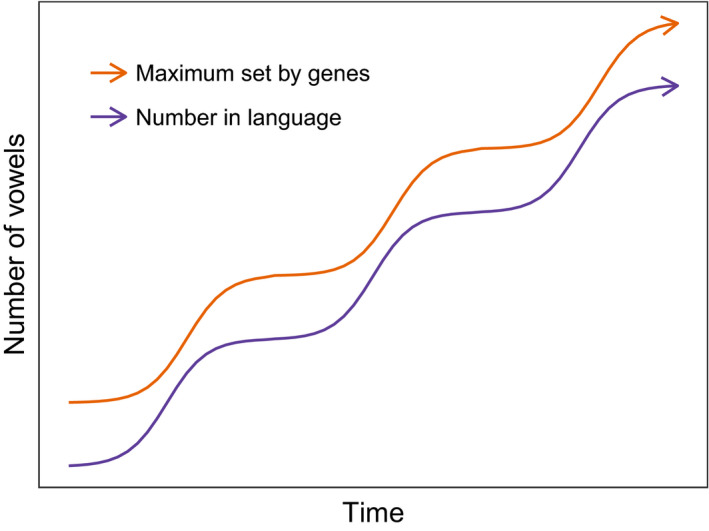
Schematic illustration of the results from de Boer (2016). The population's culture pushes at the limits of the available articulatory/perceptual space, generating selection on the genes determining the limits of that space, leading in turn to the expansion of that space and further cultural evolution.

De Boer studies the evolution of two biological capacities related to the production of vowels: the size of the overall articulatory space, as determined by, for example, the configuration of the vocal tract, and perceptual sensitivity, which might be determined by the neural circuitry involved in perception. These factors are taken to be encoded in an individual's genes and have consequences for the kind of vowel system an individual can develop; individuals with a larger articulatory space and/or a more fine‐grained perceptual sensitivity can sustain vowel systems involving more discriminable vowels (either because the available space is larger, or the available space can be filled more densely with vowels which can nonetheless be discriminated). Under the further assumption that better communicators are more likely to reproduce and pass on their genes to the next generation, de Boer shows a niche construction dynamic where the vowel system pushes continuously at the outer limits of the available articulatory or perceptual space, generating a selection pressure favoring individuals whose genes give them a larger vowel space or finer grained perceptual abilities; as these individuals take over the population, cultural evolution again pushes out the vowel system to take advantage of the new space, generating further selection for ever‐more‐able individuals (see Fig. 4). Similar models applied to the capacities for learning compositional languages might show the evolution of increasingly sophisticated abilities for acquiring or exploiting such systems, once the initial co‐evolutionary process was established.

This process of gene–culture co‐evolution might also act to *constrain* cultural evolution, by imposing biological constraints on the kinds of systems which can be learned. This possibility is of particular relevance in the evolution of syntax, where several prominent theories assume that there are constraints on the form of grammars that can be acquired, including in some theories constraints which appear arbitrary with respect to function (e.g., Chomsky, 1965). While recent versions of these theories have sought to move the source of such constraints beyond the core linguistic system (Chomsky, 2005), evolutionary explanations for constraints on linguistic variation have been offered; for example, “the requirement for standardisation of communication protocols dictates that … many grammatical principles and constraints must accordingly be hardwired into the [language acquisition] device” (Pinker & Bloom, 1990, p. 720).

This perspective has received some support from co‐evolutionary modeling. Nowak, Komarova, and Niyogi (2001) model the evolution of constraints on the size of the set of languages considered by learners: more constrained learners are only capable of learning languages drawn from a smaller set. They provide two general results. First, there is selection in favor of a language faculty which restricts the number of possible languages to levels that allow a population to converge on a shared grammar, as predicted by Pinker and Bloom (1990). However, selection will not lead to the most constrained possible learner, since there are costs to being overly constrained (i.e., inability to acquire one of several languages in use in the population). This balance of selective pressures yields learners whose language faculty is permissive, allowing them to learn the largest possible set of languages, but nonetheless constraining enough to permit convergence within a population. This suggests a co‐evolutionary process similar to that demonstrated by de Boer (2016); in both cases, culture introduces a selection pressure and selection tunes the properties of individuals to match that pressure. Similar results are provided by, for example, Kirby and Hurford (1997) and Briscoe (2000): Stable features of linguistic systems produced by cultural evolution are assimilated into the genome, suggesting that gene–culture co‐evolution might lead to evolved constraints on language learning.

#### 3.2.2 The moving target problem

This work therefore suggests a rather harmonious relationship between biological and cultural evolution. However, Chater, Reali, and Christiansen (2009) note that these models assume that the relevant features of the linguistic environment are stable. They argue that linguistic features which change more rapidly (perhaps because they are arbitrary with respect to function, and therefore not constrained *not* to change) are less likely to drive selection. In other words, if language constitutes a “moving target” for the genes, then features which change rapidly enough will not to be assimilated into the genome.

**Figure 5:**
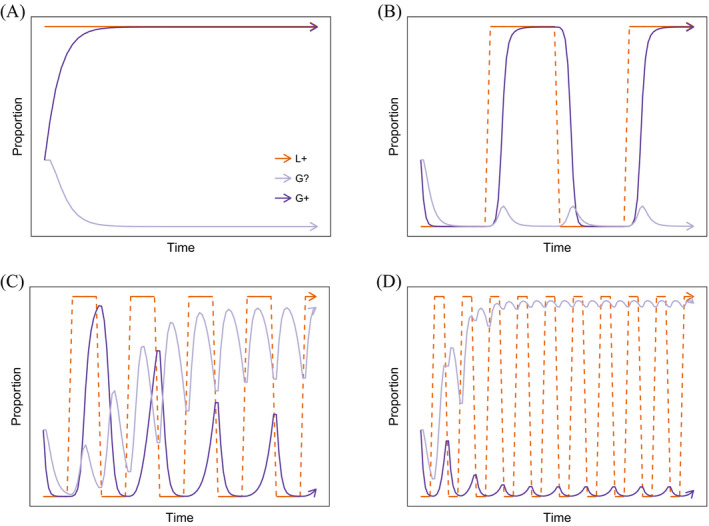
Numerical simulation of a simplified version of the moving target model from Chater et al. (2009). When the language is stable or changes only very slowly (A,B), constraints on learning reflecting that stable linguistic environment are selected for (here, the evolution of the G+ gene is shown). However, when the linguistic environment changes rapidly (C,D), such constraints do not evolve; the G+ gene is selected against, and neutral G? individuals proliferate.

They support this claim with models of the evolution of constraints on language learning against a background of a more‐or‐less fluctuating linguistic feature (Baronchelli, Chater, Christiansen, & Pastor‐Satorras, 2013; Chater et al., 2009). Chater et al. (2009) imagine that linguistic features can take one of two values, L+ and L−. Individuals have three possible predispositions toward that linguistic feature, encoded in a single gene: Individuals with the G+ gene find it easy to acquire the L+ variant, and hard to acquire the L− variant; conversely, individuals with the G− gene find it hard to acquire the L+ feature and easy to acquire the L− feature; individuals with the neutral G? gene have intermediate difficulty in acquiring either linguistic variant; that is, they are slower to acquire the L+ variant than a G+ individual, but faster to acquire it than a G− individual. If the linguistic environment is fixed, and on the assumption that faster learning has a fitness payoff (i.e., faster learners have more offspring), the genes matching that linguistic environment increase in frequency, because learners with the right gene learn the language faster (see Fig. 5A,B). However, even modest rates of language change (modeled as flips from one linguistic feature to another) result instead in neutral genes being selected for (see Fig. 5C,D). This contributes to what Christiansen and Chater (2008) call the “logical problem of language evolution,” casting doubt on the possibility that a set of domain‐specific arbitrary constraints (as envisaged under some linguistic theories) could be produced by evolution.

**Figure 6:**
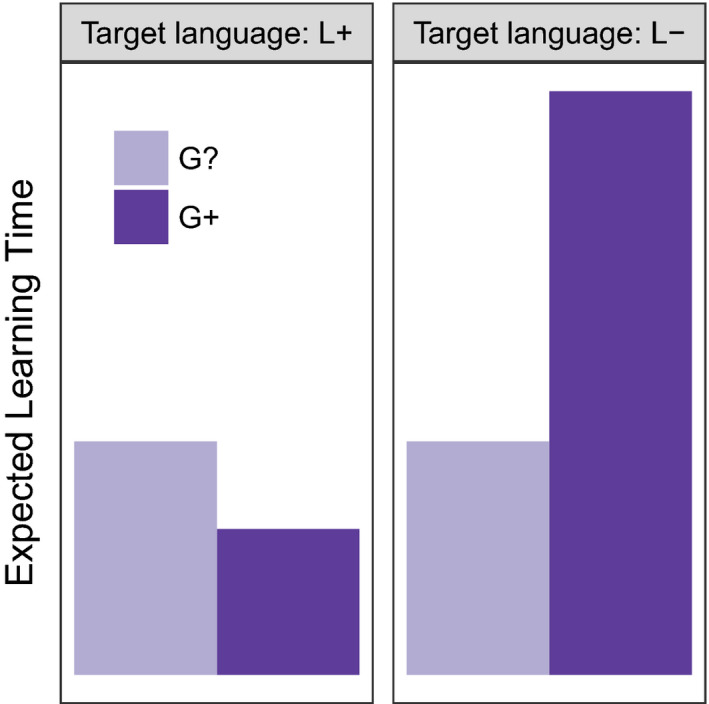
The moving target model as implemented in Chater et al. (2009) assumes that the disadvantages that specialist learners face when learning languages of the dispreferred type are greater than the advantages they have when learning languages of the preferred type. Chater et al. (2009) model learning as a trial‐and‐error process where the learner, aiming to match a target linguistic feature, samples a value randomly, with the bias on sampled features determined by the gene. An individual with the G? gene trying to match linguistic feature L+ will sample L+s or L−s with equal probability until they hit a L+ feature, at which point learning is finished. G? individuals can therefore be expected to take two exposures to learn either the L+ or L− feature. Individuals with a G+ gene are far more likely to sample a G+ feature (e.g., G+ individual samples the L+ feature with probability 0.8, 0.95, or 1; results in this plot are for a bias of 0.8). Biased individuals have a slight speed advantage in learning the linguistic feature that matches their genetic preference (e.g., expected learning time of 1.25 trials here), but they suffer a larger disadvantage when trying to learn the dispreferred feature (expected learning time of 5 here). For the strongest bias (probability of sampling the preferred feature = 1) the biased learner learns the preferred language twice as fast as an unbiased learner but can *never* learn the dispreferred language.

However, recent modeling work (de Boer & Thompson, 2018) suggests that Chater et al. (2009) might have been too pessimistic in their assessment of the potential for evolution to track a changing linguistic environment. First, they note that the model of genetic specialization used by Chater et al. includes far greater penalties than benefits for individuals whose genes encode a predisposition to acquire a particular linguistic feature (see Fig. 6). Language change therefore generates stronger selection *against* specialization than positive selection for it. Second, de Boer and Thompson (2018) show that, after removing this disadvantage to genetic specialization, the extent to which the moving target problem prevents innate constraints evolving depends on other factors, including the size of the population and the extent to which a small number of biased individuals have a large effect on language change (e.g., by making the language more likely to change to the preferred state, and unlikely to change away from it); for a wide range of parameter settings, they find that moderate rates of language change do not radically alter the probability of specialist genes going to fixation, contrary to the moving target hypothesis. Whether we should expect gene–culture co‐evolution to produce innate constraints on the learning of a particular linguistic feature therefore depends on how rapidly we expect that feature to change through cultural evolution, how independent those cultural changes are of the population's genetic composition, and to what extent the evolution of the population's genetic and linguistic system is determined by fitness rather than stochastic effects arising in small populations.

#### 3.2.3 Unmasking, masking, and the strength of constraints on learning

Setting the moving target problem aside, another recent paper also suggests more subtle interactions between culture and biology in shaping linguistic systems. Building on earlier work by Smith and Kirby (2008), Thompson, Kirby, and Smith (2016) model co‐evolution in a population of rational (Bayesian) learners who come to the language learning task with a prior bias which, in combination with their linguistic data, shapes their eventual linguistic system. Similar to Chater et al. (2009), each individual's bias is assumed to be encoded in a series of genes which are inherited from their parent; opportunities for reproduction are proportional to the probability of sharing the same language type as other members of the population, building in an assumption that linguistic parity is required for communication (as envisaged by e.g., Pinker & Bloom, 1990), rather than assuming speed of learning is the determining factor. In the simplest version of this model, every individual can be characterized by a single linguistic feature which can take two values, L+ or L−; each individual's genotype consists of multiple (e.g., 100) genes which encode the learner's bias with respect to this linguistic feature (the learner's bias in favor of the L+ feature is given by the proportion of their genotype made up of G+ genes). Learners infer a language type based on the linguistic data they observe and their genetically determined prior bias, via the process of Bayesian inference; we assume that learners select the most probable language based on their data and biases, that is, the maximum a posteriori (MAP) language.

**Figure 7:**
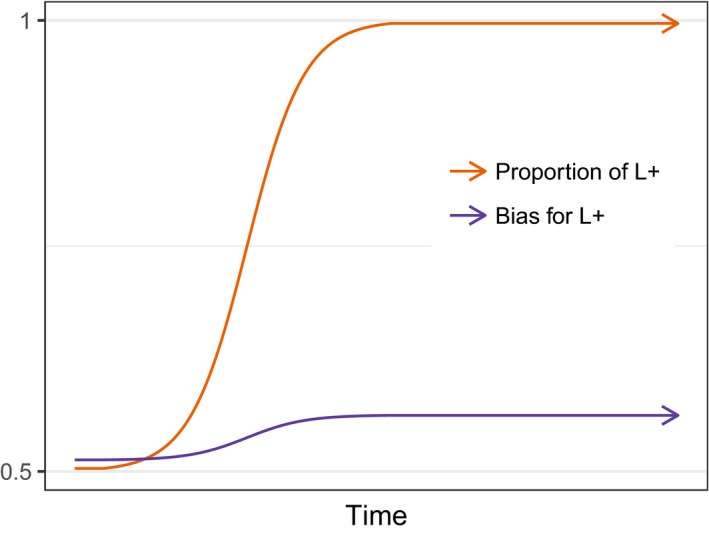
Schematic illustration of the results from Thompson et al. (2016), showing proportion of speakers of the L+ language in the population, and the genetically encoded prior bias toward L+. A slight bias in the population's initial genes, favoring L+, leads to the rapid evolution of the L+ language and the development of a linguistic universal (unmasking). This emerging universal leads to rapid evolution of a weak learning bias in favor of L+, but that bias never becomes a strong constraint (due to masking).

The results of co‐evolution in this model reveal a distinct pattern, unlike either niche construction or the moving target model (see Fig. 7). First, even a slight bias in favor of one of the two language types (arising e.g., from random fluctuations in the frequency of G+ genes) leads the population's language to rapidly converge on that language type; small biases in individuals are *unmasked* and amplified by culture. This amplification of biases is a known consequence of cultural transmission under a fairly wide range of circumstances (Boyd & Richerson, 1985; Griffiths & Kalish, 2007; Kirby, Dowman, & Griffiths, 2007). Unmasking generates a substantial selection pressure for genes that bias individuals in favor of learning the to‐be‐dominant language type; in the event that an individual receives linguistic data which is ambiguous as to which language they should learn, a nudge in the right direction from their genes is advantageous. However, as the population's culture converges on a single shared language type, learners are increasingly presented with linguistic data that provides strong evidence for the right language: In a population with a small majority of L+ speakers, a bias in favor of the L+ language is helpful, but in a population where only the L+ language is present, every individual can rely on receiving data that leads them to learn the L+ language. Convergence by cultural evolution, in combination with the properties of MAP learning, means that there is therefore little advantage to strong biases—any bias in the right direction will do. The strength of bias in individuals is therefore *masked* from selection: Individuals with rather different genotypes tend to learn the population's language reliably and are therefore equally likely to be selected to reproduce. When unmasking and masking are at play, a weak bias in favor of the culturally evolved linguistic universal emerges extremely rapidly (thanks to unmasking) but that weak bias never turns into a strong constraint on learning (due to masking): Culture facilitates the rapid evolution of weak biases in learning but also prevents the evolution of strong constraints. Note importantly that none of the models discussed above include both the unmasking and masking processes on which this result depends; in Chater et al. (2009) and de Boer and Thompson (2018) fitness depends on speed or accuracy of learning, which, in their models, directly and transparently reflects an individual's genes, and hence genes are never masked from selection.

The co‐evolutionary picture for language therefore depends on what we think the precise relationship between genes and culture is—and this relationship may differ for different aspects of the linguistic system. For cases where masking and unmasking do not operate, we might see a scenario where biological evolution of linguistic capacities interacts with the cultural evolution of the linguistic system, with culture pushing the envelope of possible systems and biology expanding the available space for culture to operate (as shown by de Boer, 2016), or acting to nativize stable constraints on linguistic systems (as shown in Chater et al., 2009; de Boer & Thompson, 2018; Nowak et al., 2001, notwithstanding the moving target problem). In contrast, where unmasking and masking dynamics both operate—where weak biases in individuals can have a strong effect on the population's linguistic system, and individuals immersed in the right kind of linguistic data have learning outcomes rather independent of any biases in their genes—we should expect weak biases on learning to evolve rapidly, but never become strong constraints. These co‐evolutionary models therefore suggest that the human capacity for language might be a mosaic of traits, produced by several rather distinct co‐evolutionary processes.

### 3.3 Toward a richer model of the co‐evolution of language and cognition

All of the foregoing co‐evolutionary modeling assumes that the capacities learners bring to language learning are genetically determined and fixed, unmodified by language learning experience. This assumption looks increasingly untenable for language learning, where the biases and capacities that shape learning from linguistic data are themselves shaped by linguistic experience. For example, learning words allows children to extract generalizations about words which facilitate subsequent word learning. The *shape bias* is a classic example of such a higher‐order generalization, which applies to many nouns in English: Older children and adults assume that words generalize by shape rather than some other property of their referents, for example, size or texture. This bias develops over time in English‐speaking children (Landau, Smith, & Jones, 1988), its development can be accelerated with structured training (Smith, Jones, Landau, Gershkoff‐Stowe, & Samuelson, 2002), and the strength of the bias in individuals relates to the composition of their lexicons (children whose lexicons are more consistent with the shape bias show a stronger shape bias: Perry & Samuelson, 2011), suggesting it is a generalization that children extract from linguistic data. Similar generalizations can be made in other lexical domains (e.g., appropriate training modulates higher‐order generalizations about whether verbs encode manner or path of motion: Shafto, Havasi, & Snedeker, 2014), and linguistic experience shapes other word learning biases (e.g., bilingual children show a smaller mutual exclusivity bias than monolinguals, presumably reflecting their experience that objects can have multiple labels: Houston‐Price, Caloghiris, & Raviglione, 2010); the very fact that most symbolic labels in spoken languages take the form of words (rather than simply any kind of sound) may itself be a generalization over experience with symbols (Thom & Sandhofer, 2014). Furthermore, this kind of reciprocal bootstrapping does not only occur within the linguistic system. As reviewed in Woensdregt and Smith (2017), exposure to a natural language facilitates development of skills involved in reasoning about mental states in others, which in turn play a role in language learning: Individuals with Theory of Mind impairments suffer language learning deficits (Parish‐Morris, Hennon, Hirsh‐Pasek, Golinkoff, & Tager‐Flusberg, 2007), and children's development of Theory of Mind is delayed when they cannot access the linguistic system of their community (e.g., as is the case for deaf children of hearing parents, Schick, de Villiers, de Villiers, & Hoffmeister, 2007) or when that community's language is still in the process of evolving ways to communicate about mental states (as in the case of Nicaraguan Sign Language, where signers from early cohorts show impaired reasoning about mental states of others: Pyers & Senghas, 2009).

While models of hierarchical learning in individuals are available (e.g., Kemp, Perfors, & Tenenbaum, 2007; Regier, 2003; Thompson & de Boer, 2017), evolutionary models are currently lacking. As suggested by Thompson and de Boer (2017), it may be that selection has enhanced the human capacity to form higher‐order generalizations. Co‐evolutionary models which extend the work of, for example, Thompson et al. (2016) or de Boer (2016) to the case of hierarchical learning could evaluate the evolutionary feasibility of this kind of co‐evolution, and comparative studies of the propensity of humans and other animals to extract higher‐order generalizations (such as the shape bias) would provide direct evidence as to whether humans have an unusual propensity to learn in this way. But it may be that purely cultural processes can shape higher‐order learning and perhaps even the capacities underpinning the cultural evolution of structure. A first step would be to show the cultural evolution of higher‐order generalizations, exploring whether and when an incipient bias (e.g., to generalize object labels by shape) reshapes the lexicon in such a way that that bias is strengthened in individuals at subsequent generations, leading to further sharpening of the signal for that bias in the lexicon. More ambitiously, the same techniques could be used to explore whether the capacities required for the cultural evolution of structured language (ability to learn signals, infer communicative intentions, and acquire structured form‐meaning mappings) can bootstrap themselves into existence through purely cultural mechanisms, as the evolving language fosters the (ontogenetic, rather than evolutionary) development of the capacities required to acquire and extend that cultural system.

## 4 Conclusions

Languages evolve as a result of their learning and use, and that process of cultural evolution shapes the evolution of the capacity for language. If we are interested in how culture shapes the evolution of cognition (for language or other behaviors), we need to understand both how cognition shapes culture, and how this in turn allows culture to reshape cognition, either through gene–culture co‐evolution or acquired biases in learning. Existing work from evolutionary linguistics shows that biases in cognition and communication shape linguistic systems and offers an explanation for some of the fundamental structural properties of language; co‐evolutionary work shows how this process might in turn drive the evolution of those cognitive capacities. The evolution of linguistic systems therefore provides a fascinating test case for exploring how biology and culture interact on evolutionary timescales to shape cognition.

## Note

1 There may be cases where the set of distinct communicative signals that can be produced is very large or even open‐ended, as in cases where complex vocalizations are built by combining elements from a smaller inventory of recombinable units, for example, in birdsong or the song of humpback whales (Catchpole & Slater, 1995; Payne & McVay, 1971). However, combinatoriality in these song systems is (as far as we currently know) essentially ornamental and serves no referential function; in other words, such systems are combinatorial but not compositional, and the open‐endedness of the set of signals therefore does not subserve open‐ended semanticity in the same way as in human language.
